# The level of specific IgE is a moderate predictor for the outcome of a double-blind placebo-controlled food challenge for hazelnut in children

**DOI:** 10.1186/2045-7022-1-S1-O37

**Published:** 2011-08-12

**Authors:** Laury Masthoff, Suzanne Pasmans, Mirjam Knol, Els van Hoffen, Annebeth Flinterman, Petra Kentie, André Knulst, Yolanda Meijer

**Affiliations:** 1 University Medical Center Utrecht, (Pediatric) Dermatology/Allergology, Utrecht, Netherlands; 2 University Medical Center Utrecht, (Pediatric) Dermatology/Allergology, Center of Pediatric Allergology, Wilhelmina Children's Hospital, Pediatric Pulmonology, Utrecht, Netherlands; 3 University Medical Center Utrecht, Julius Center for Health Sciences and Primary Care, Utrecht, Netherlands; 4 University Medical Center Utrecht, Center of Pediatric Allergology, Wilhelmina Children's Hospital, Pediatric Pulmonology, Utrecht, Netherlands

## 

Literature about the value of diagnostic tests for hazelnut allergy in children is scarce. For peanut allergy cutoff levels of specific IgE with a 95% positive predictive value (PPV) were published. To evaluate current diagnostics for hazelnut allergy in children, data of 151 children, who underwent a double-blind placebo-controlled food challenge (DBPCFC) for hazelnut were analyzed. The PPV or negative predictive value (NPV) of the level of specific IgE (CAP) for hazelnut and the size of the skin prick test (SPT) for hazelnut was determined. The influence of spiking of the CAP for hazelnut with rCor a 1 was analyzed. The level of specific IgE for hazelnut was a moderate predictor for a positive DBPCFC for hazelnut. No cutoff levels of specific IgE for hazelnut with a 95% PPV could be determined. Before Cor a 1 spiking the maximum reached PPV was 73% for a cutoff level of 26 kUA/L, after spiking the maximum reached PPV was 64% for a cutoff level of 31 kUA/L. The spiking increased the NPV from 91% to 100% for a cutoff level of 0.35 kUA/L. SPT was a better predictor for a positive DBPCFC compared to the level of specific IgE. When the SPT >16 mm, the PPV was 100%. By combining both tests, the PPV reached 100% when the level of specific IgE for hazelnut was >5 kUA/L and the level of SPT was >12mm. However, the PPV of 100% for SPT alone and the combination of CAP and SPT accounted for only 11% respectively 13% of the children undergoing a DBPCFC for hazelnut. So, the level of specific IgE and reactivity of SPT are moderate predictors for the outcome of a DBPCFC for hazelnut in children. New diagnostic tools are needed to replace the DBPCFC which is burdensome, expensive and limited available.

